# Arabic Wellness Apps in the MENA Region and Saudi Arabia: Current Evidence and Systematic Evaluation

**DOI:** 10.3390/healthcare14111496

**Published:** 2026-05-28

**Authors:** Jwaher A. Almulhem, Raniah N. Aldekhyyel

**Affiliations:** Medical Informatics and e-Learning Unit, Medical Education Department, College of Medicine, King Saud University, Riyadh 12372, Saudi Arabia; raldekhyyel@ksu.edu.sa

**Keywords:** wellness apps, mHealth, Arabic, Saudi Arabia, MENA countries, ABACUS

## Abstract

**Background/Objectives**: Recent advancements in digital health have facilitated the expansion of mobile health (mHealth) apps. This study examines the top-ranked Arabic wellness mHealth apps in the Middle East and North Africa (MENA) region and evaluates wellness apps in Saudi Arabia for their potential to promote health-related behavior change using the App Behavior Change Scale (ABACUS). **Methods**: A cross-sectional review was conducted using a systematic three-phase approach to identify and evaluate Arabic wellness apps, with the dataset extracted from the Sensor Tower platform. In Phase 1, apps were identified based on predefined country, language, and wellness criteria. In Phase 2, app descriptions were screened for behavior change features. In Phase 3, eligible apps available in Saudi Arabia were downloaded and evaluated using the ABACUS. **Results**: Egypt had the highest number of identified Arabic wellness apps (*n* = 9), followed by Iraq and the United Arab Emirates (UAE) (*n* = 8 each), while Yemen had the fewest identified apps. For potential behavior change apps, Tunisia and the UAE had the highest apps (*n* = 7), whereas Yemen and Libya had the lowest (*n* = 1 each). All Arabic wellness apps identified in Saudi Arabia were classified as promoting physical activity with only one app encouraging healthy eating. The total ABACUS scores were relatively consistent, ranging from 15 to 17 out of 21. **Conclusions**: Arabic mHealth wellness apps identified across the included MENA countries were limited in number, while apps available in Saudi Arabia showed variability in the incorporation of behavior change features.

## 1. Introduction

Recent advancements in digital health and the widespread adoption of smartphones have driven substantial growth in the mobile health (mHealth) app market [1,2,3]. These apps are increasingly shaping how care is delivered and how individuals manage their health [4]. Wellness apps, in particular, have expanded rapidly in recent years and are expected to continue expanding [5]. These apps offer users personalized features for health monitoring, behavior change support, and disease management [6]. A further advantage of mHealth apps is their accessibility. Unlike traditional healthcare services, they can reach users beyond clinical settings, reducing financial and logistical barriers to care, which makes them especially relevant in contexts where healthcare access is limited [7,8]. Beyond accessibility, growing evidence suggests that mHealth interventions can promote positive health behavior change, with studies reporting improvements in physical activity levels and reductions in sedentary behavior [9,10].

Similar trends are evident in the Middle East and North Africa (MENA) region. Growing smartphone adoption, particularly in urban areas, has created a favorable environment for mHealth solutions, especially Arabic-language apps [11]. However, the increasing availability of such solutions has not been accompanied by consistent evidence regarding their quality or effectiveness. Existing apps vary considerably in their design, use of evidence-based approaches, and ability to support sustained behavior change [12].

Research examining mHealth interventions across various settings has identified several factors that influence their effectiveness. Beyond technical performance, these factors include user engagement, incorporation of behavior change strategies, and the provision of accurate and usable health information [13,14]. Studies examining physical activity and weight management apps have further suggested that apps with higher behavior change scores are more likely to support user engagement and sustained behavior change [15,16]. In particular, features such as goal setting, self-monitoring, feedback, and social support appear to play an important role in achieving these outcomes [6,17,18].

Given this complexity, standardized evaluation tools have become increasingly important for assessing app quality [18,19]. One widely used instrument designed specifically to examine the behavioral components of mHealth apps is the App Behavior Change Scale (ABACUS) [20]. Developed by McKay et al. [20] through a comprehensive review of behavior change frameworks and expert consensus, ABACUS is a validated tool that assesses whether mHealth apps incorporate established behavior change techniques with the potential to influence user behavior [20,21]. Initial validation across 70 physical activity apps demonstrated strong interrater reliability and construct validity [20].

Understanding app quality is especially critical in regions where the burden of lifestyle-related disease is high. The MENA region, for instance, has seen rising rates of obesity and physical inactivity, both of which contribute to increasing prevalence of type 2 diabetes and cardiovascular disease [22,23]. In Saudi Arabia, these conditions remain highly prevalent across different age groups, reflecting a broader regional pattern [24]. Access to preventive and lifestyle-focused services, however, is not always consistent, with persistent barriers related to geography, service availability, and healthcare system capacity [25]. Addressing these challenges aligns with the objectives of Saudi Vision 2030 [26], which prioritizes prevention, early intervention, and population well-being [27]. Through initiatives such as the Health Sector Transformation Program [28], national efforts continue to promote healthier lifestyles and support the integration of digital health solutions into healthcare delivery.

However, despite the growing number of mobile apps targeting physical activity, fitness, and weight management among Arabic-speaking users [11], the evidence base surrounding these tools remains limited. Much of the existing literature has focused on describing available apps rather than systematically evaluating them. The degree to which such apps incorporate evidence-based behavior change features remains poorly understood [29,30]. Most evaluation studies to date have focused primarily on English-language apps, often overlooking the cultural and linguistic context of users in the MENA region [4,5,6,7]. Few studies have systematically evaluated Arabic wellness apps using validated assessment frameworks or examined their potential to support behavior change among Arabic-speaking populations [31,32].

To address these gaps, this study aimed to identify top-ranked Arabic wellness apps across the MENA region and evaluate a subset of apps available in Saudi Arabia using the ABACUS scale [20]. Applying this standardized behavior change framework provides a snapshot of the current landscape of Arabic mHealth wellness apps. The findings may inform the development of future apps that better incorporate evidence-based behavior change features.

## 2. Materials and Methods

### 2.1. Study Design

A cross-sectional review was conducted to evaluate Arabic wellness apps available in the MENA region. Apps were identified and the dataset was extracted using the Sensor Tower analytics platform (Sensor Tower Inc., San Francisco, CA, USA) [33]. Eligible apps, which included behavior change features available in Saudi Arabia were evaluated using the ABACUS framework [20].

### 2.2. ABACUS Framework Description

The ABACUS consists of 21 items scored using a binary format, where each item is rated as absent (0) or present (1) based on the inclusion of the corresponding behavior change feature within the app. Scores are summed to generate a total behavior change score for each app. The items are organized into four domains: knowledge and information (5 items), which assess the provision of health-related instructions and information; goals and planning (3 items), which evaluate features such as goal setting and planning for social support; feedback and monitoring (7 items), which examines functions related to self-monitoring and performance feedback; and actions (6 items), which assesses features that encourage behavioral practice and reinforcement [20].

The ABACUS was chosen as it offers a standardized, evidence-based framework for assessing behavior change techniques in wellness apps, consistent with the study’s primary objective. The instrument’s scope, which does not extend to domains such as usability, readability, privacy, security, or clinical validity, was appropriate given this focus.

### 2.3. App Selection

**Figure 1:**
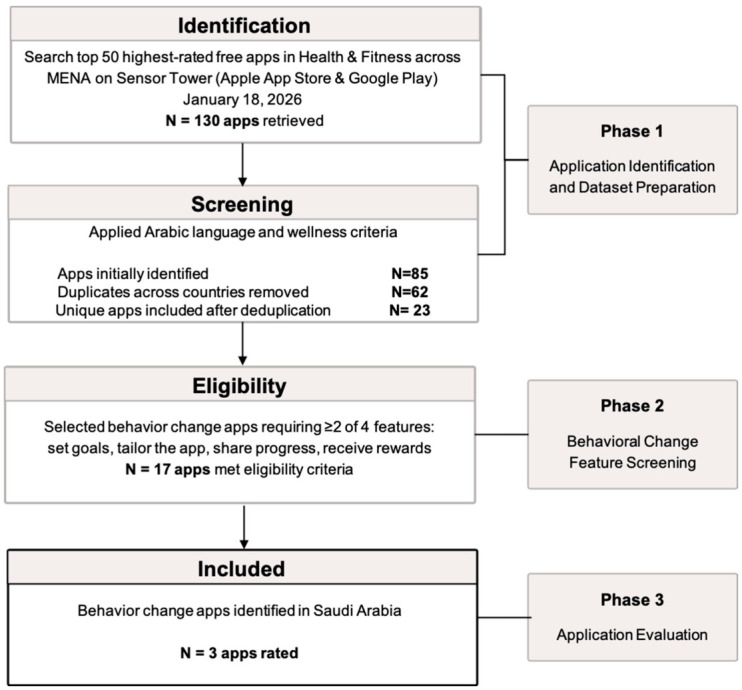
Application Screening and Evaluation Process.

A systematic three-phase approach was used to identify and select Arabic wellness mHealth apps. The definition of wellness apps was established following a review of three national documents: (1) the Ministry of Health Digital Strategy Framework and Roadmap [34], (2) the Saudi Food and Drug Authority (SFDA) guidelines [35], and (3) the Public Health Authority documents [36]. Among the definitions reviewed, that of the SFDA was the most inclusive, defining a wellness app as “a mobile app that promotes physical activity, healthy eating, stress management, sleep, and smoking cessation” [35]. This definition was then used to guide the extraction of the dataset from Sensor Tower [33], which was then screened across three phases: (1) app identification and dataset preparation, (2) behavioral change feature screening, and (3) app evaluation using the ABACUS framework (Figure 1).

Throughout all three phases, screening and evaluation were conducted collaboratively by the research team through consensus-based meetings to ensure consistency in the interpretation and application of criteria across all apps.

#### 2.3.1. Phase 1: App Identification and Dataset Preparation

On 18 January 2026, the study dataset was extracted from the Sensor Tower analytics platform [33] across three sequential stages: (1) app identification and duplicate removal, (2) screening for Arabic language support, and (3) identification of wellness apps according to the SFDA definition [35].

In the first stage, searches were conducted separately within the Apple App Store (Apple Inc., Cupertino, CA, USA) and Google Play Store (Google LLC, Mountain View, CA, USA) for each included MENA country using the Sensor Tower platform. The search filters applied included: (1) app category (“Health and Fitness”), (2) ranking type (“Free”), and (3) country-specific app store selection. Countries were selected based on the World Population Review classification of the MENA region [37] which included Algeria, Bahrain, Egypt, Iraq, Jordan, Kuwait, Lebanon, Libya, Morocco, Oman, Qatar, Saudi Arabia, Tunisia, the United Arab Emirates (UAE), and Yemen. Syria and Palestine, although Arabic-speaking, were excluded due to insufficient data availability on Sensor Tower. For each country-platform combination, the top 50 ranked free apps identified on 18 January 2026, were extracted for further analysis. To ensure each app was represented only once in the final dataset, duplicates across platforms and countries were identified by cross-referencing app names and developer information. Where uncertainty existed, app icons, descriptions, and publisher details were manually reviewed, with apps appearing across multiple stores or country rankings counted only once in the final dataset.

In the second stage, extracted apps were screened for Arabic language support to ensure cultural and linguistic relevance for MENA users. Apps with unclear language availability or regional accessibility were resolved through manual review of app store descriptions.

In the final stage, wellness apps were identified according to the SFDA definition [35].

#### 2.3.2. Phase 2: Behavior Change Feature Screening

App descriptions were screened for the presence of four predefined behavior change features adapted from Middelweerd et al.’s research [38]: (1) goal setting, defined as the ability to establish specific and measurable health-related targets, such as daily step goals; (2) app tailoring, whereby users could personalize features or recommendations according to their preferences or needs; (3) progress sharing, through social media platforms or in-app community features; and (4) rewards or acknowledgments, including badges, points, congratulatory messages, or other forms of positive reinforcement following goal achievement.

Each feature was scored as either present or absent based on information available within the app description. Goal setting was considered present if users could enter and monitor daily targets; app tailoring was identified when customized workout plans were generated; progress sharing was considered present when achievements could be communicated to others; and rewards were identified when the app provided badges, milestone notifications, or congratulatory feedback [38].

Apps meeting at least two of the four criteria were included for further evaluation, consistent with the approach described by McKay et al. [39].

#### 2.3.3. Phase 3: App Evaluation

All eligible behavior change apps available in Saudi Arabia that did not require Apple ID sign-in were downloaded onto an iPhone device. Each app was explored by the research team for approximately 15 min to ensure familiarity with its features and functionality. During this process, key app components were systematically reviewed, including onboarding procedures, goal setting, personalization, activity tracking, feedback mechanisms, social sharing functions, and reward systems.

In addition to the ABACUS assessment, technical app characteristics were documented, including requirements for add-ons or in-app purchases, presence of advertisements, privacy statements, password protection, data export functionality, sharing capabilities, community features, login requirements, reminder settings, web access requirements, and push notification permissions, following the methodology described by McKay et al. [39].

To facilitate standardized data collection, a Google Form was developed consisting of two sections: (1) app characteristics and technical features, and (2) the 21 ABACUS items. Scoring was conducted collaboratively by the research team through consensus-based evaluation meetings, during which all apps were jointly reviewed and assessed (Appendix A).

### 2.4. Data Analysis

Descriptive statistics were used to characterize the identified apps. Frequencies and percentages were reported for categorical app features and functionalities, while means were calculated for continuous variables. The total ABACUS score was calculated by summing the individual item scores, each rated as either present (1) or absent (0), yielding a maximum possible score of 21. Mean ABACUS scores were subsequently calculated across included apps. All analyses were performed using Microsoft Excel version 365 (Microsoft Corp., Redmond, WA, USA).

## 3. Results

Following the screening and eligibility assessment, 23 unique Arabic wellness apps were identified across MENA countries, of which 17 met the predefined behavior change criteria (Appendix B). Four Saudi Arabian apps were eligible for ABACUS assessment; however, one app was excluded as it required an Apple ID sign-in. Consequently, three apps were included in the final evaluation.

As shown in Table 1, Egypt had the highest number of Arabic wellness mHealth apps (*n* = 9), followed by Iraq and the UAE (*n* = 8 each). In contrast, Yemen had the lowest number of identified Arabic wellness apps. Regarding behavior change apps, Tunisia and the UAE had the highest numbers (*n* = 7 each), whereas Yemen and Libya had the lowest representation.

Table 2 presents the categories, user ratings, and technical characteristics of the three apps identified in Saudi Arabia. All three focused on promoting physical activity, with one additionally targeted healthy eating. Two of the three apps had a user rating of 4.7.

All three apps could be used without additional add-ons, included password protection, featured an in-app community, required user login, provided reminder notifications, and requested permission for push notifications. None required a one-time purchase or continuous web access for functionality. In-app advertisements, privacy statements, and the ability to export or share data were identified in two of the three apps.

All three eligible apps identified in Saudi Arabia were evaluated using the ABACUS framework. Total scores were relatively similar across apps, ranging from 15 to 17 out of a maximum score of 21 (Table 3). The mean ABACUS score was 16, indicating substantial incorporation of behavior change techniques across the three identified apps.

Within the knowledge and information domain, most behavior change techniques were present across all apps, with the exception of features related to expert involvement in app development and provision of information regarding behavioral consequences. Similarly, most techniques within the goals and planning domain were identified across the evaluated apps; however, assessment of users’ willingness to change behavior was present in only one app.

**Figure 2:**
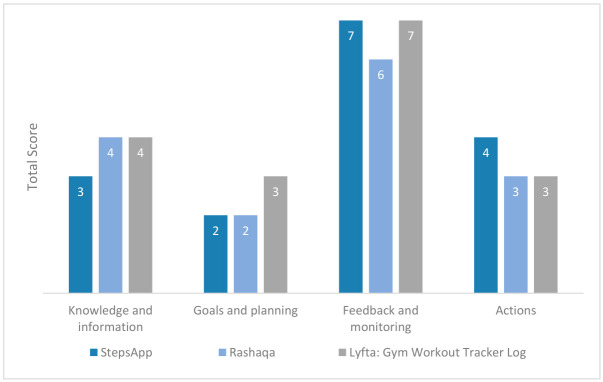
Total score for each app based on the ABACUS domains.

In the feedback and monitoring domain, nearly all techniques were identified across the three apps, with the exception of data export functionality, which was absent in one app. Within the actions domain, three techniques were present across all apps, while two techniques, namely restructuring the physical or social environment and assistance with distraction, were absent in all evaluated apps. Detailed domain-level findings are presented in Figure 2 and Table 3.

## 4. Discussion

This study explored the availability of Arabic mHealth wellness apps in the MENA region and evaluated a subset of Saudi Arabian apps using the ABACUS framework [20]. A key finding was the limited number of available Arabic wellness apps despite the growing regional emphasis on digital health, prevention, and population wellness [27]. Although national programs such as Saudi Vision 2030 prioritize lifestyle improvement and preventive health [28], the identified apps may not yet fully reflect these policy ambitions, suggesting challenges in translating national digital health strategies into accessible, user-centered technologies.

This limited availability may indicate structural and ecosystem-level barriers to digital health innovation across parts of the MENA region. Differences in app availability across countries likely reflect disparities in digital infrastructure, economic stability, investment capacity, and digital health maturity. Countries such as Egypt and the UAE demonstrated greater app availability, whereas Yemen and Libya had substantially fewer identified apps. Such disparities may influence equitable access to digital preventive health resources across the region [40,41].

Beyond infrastructure and investment disparities, the limited app availability may also reflect broader regulatory and market-related challenges within the regional digital health landscape. Developing evidence-based mHealth interventions requires collaboration between healthcare professionals, behavioral scientists, and technology developers, yet such interdisciplinary partnerships may remain limited in emerging digital health ecosystems [13]. Commercialization pressures may influence which apps are prioritized for development and long-term sustainability, potentially favoring market-driven fitness applications over broader public health needs.

Language accessibility also emerged as an important finding. Although Arabic language support was part of the eligibility criteria, variation existed in how language localization was implemented during onboarding and app use. In the MENA region, where users have varying levels of digital and health literacy, language accessibility is closely linked to trust, comprehension, and engagement [42]. Apps that do not prioritize culturally and linguistically tailored onboarding experiences may unintentionally limit accessibility, particularly for older adults and individuals less familiar with English-based digital interfaces [30,43,44].

Beyond app availability findings across MENA countries, evaluation of the Saudi Arabian subset using the ABACUS framework provided preliminary insights into the incorporation of evidence-based behavior change techniques. The evaluated apps primarily focused on physical activity, with only one including healthy eating features. While physical activity promotion is an important public health priority, this narrow focus may indicate limited diversification within the evaluated subset. Other key health promotion domains, including mental health and tobacco cessation, were not represented among the evaluated apps despite their established importance within regional public health agendas [35]. This imbalance may partly reflect commercialization trends favoring fitness-oriented applications, which are often easier to market and monetize. Wellness interventions targeting isolated behaviors may also offer narrower support than more integrated approaches addressing multiple behavioral risk factors simultaneously [45,46].

The evaluated Saudi apps also showed inconsistent incorporation of evidence-based behavior change techniques. Although they demonstrated relatively high ABACUS scores, they lacked key features associated with long-term behavioral support. This finding aligns with previous research showing that commercial mHealth applications frequently prioritize usability and market appeal over evidence-based behavioral frameworks [47,48]. Privacy and data governance also emerged as important considerations, as features such as privacy statements, password protection, data-sharing capabilities, and data export functions were not consistently available across all apps. Given that wellness apps routinely collect sensitive information related to health behaviors, physical activity, and lifestyle patterns, transparent privacy protections may influence users’ trust, perceived credibility, and willingness to engage with these technologies [49]. These findings may further suggest variability in the implementation of digital health governance and privacy regulations across parts of the MENA region, warranting further examination in future studies.

### 4.1. Practical Implications

The findings highlight opportunities to further strengthen the Arabic mHealth ecosystem through the development of culturally tailored, evidence-based wellness applications addressing a wider range of public health priorities, including nutrition, mental health, and tobacco cessation [41,50]. Future app development should integrate behavior change theory, expert involvement, language localization, transparent privacy practices, and stronger data governance measures to strengthen scientific rigor and support user engagement.

Public health agencies may also play an important role in advancing digital wellness interventions by supporting apps that comply with established health, privacy, and quality standards, fostering collaboration between developers and healthcare experts [49]. Continued evaluation using standardized frameworks remains essential to identify emerging trends and inform future evidence based digital health development across the MENA region.

### 4.2. Strengths and Limitations

This study has several strengths, including its focus on Arabic-language mHealth wellness apps and the use of a standardized framework to assess behavior change techniques. Nevertheless, several limitations should be acknowledged. First, the cross-sectional design limited the analysis to apps available at a single point in time, while app marketplaces are continuously evolving. Second, app identification was restricted to the top 50 ranked free apps within the Health and Fitness category across the included MENA countries, which may not fully represent the broader landscape of available wellness apps. Third, the ABACUS evaluation was limited to three Saudi Arabian apps, and findings on behavior change features should therefore be interpreted with caution, as they may not be representative of the broader Arabic wellness app landscape in Saudi Arabia or the wider MENA region.

The study also focused specifically on behavior change techniques using the ABACUS framework and did not evaluate other important domains of app quality, such as usability, engagement quality, readability, clinical validity, security, or regulatory compliance. Future research should incorporate additional evaluation frameworks and examine how mHealth wellness apps are used in real-world settings, including their influence on user engagement and health outcomes. Longitudinal studies may provide a clearer understanding of app sustainability and behavioral support over time. Qualitative or mixed-methods approaches may further yield valuable insights into cultural relevance, trust, accessibility, and engagement with Arabic mHealth wellness apps. Future work should also examine apps addressing additional public health priorities, including mental health and tobacco cessation, as well as the role of interdisciplinary collaboration in improving app quality and credibility.

## 5. Conclusions

This study identified a limited number of Arabic mHealth wellness apps across the MENA region, with variation in app availability, language accessibility, and health topic diversity. Evaluation of a small subset of Saudi Arabian apps using the ABACUS framework revealed the presence of several behavior change features, though inconsistencies were observed across app characteristics and technical features. These findings highlight opportunities for developers, policymakers, and public health organizations to strengthen the Arabic digital wellness ecosystem through the development of culturally responsive, evidence-based apps that better meet the needs of Arabic-speaking populations.

## Figures and Tables

**Table 1:** Cross-Country Distribution of Identified Apps.

Country	No. of Apps (Free on Apple App Store and Google Play Store)	No. of Arabic Wellness mHealth Apps	No. Potential Behavior Change Apps
**Saudi Arabia**	13	6	4
**Egypt**	14	**9**	6
**Algeria**	11	6	5
**Iraq**	8	**8**	5
**Yemen**	2	2	1
**Morocco**	7	5	2
**Tunisia**	9	7	**7**
**Jordan**	8	7	5
**United Arab Emirates**	**16**	**8**	**7**
**Libya**	3	2	1
**Lebanon**	10	6	4
**Oman**	9	7	5
**Kuwait**	7	4	4
**Qatar**	6	4	4
**Bahrain**	7	4	4

Values in bold indicate the highest number of apps.

**Table 2:** User Ratings and Features of the Included Potential Behavior Change Apps in Saudi Arabia.

App Name	StepsApp	Rashaqa	Lyfta: Gym Workout Tracker Log
**Category**	Increasing physical activity	Increasing physical activity and promoting healthy eating	Increasing physical activity
**Developer**	StepsApp GmbH	Madar Al Parmaga	Lindberg Development AS
**Version**	8.7.3	17.5	1.140
**User rating**	4.7	4.5	4.7
**Can be used without add-ons**	Yes	Yes	Yes
**Requires in-app payments**	Yes	No	No
**One-off purchase required**	No	No	No
**Had in-app advertisements**	Yes	Yes	No
**Had a privacy statement**	Yes	No	Yes
**Allowed password protections**	Yes	Yes	Yes
**Allowed data to be exported**	Yes	No	Yes
**Allowed sharing**	Yes	No	Yes
**Had an app community**	Yes	Yes	Yes
**Required login**	Yes	Yes	Yes
**Sent reminders**	Yes	Yes	Yes
**Needed Web access to function**	No	No	No
**Asked permission for push notifications**	Yes	Yes	Yes

**Table 3:** Comparison of Apps Based on the ABACUS Items.

ABACUS Item	StepsApp	Rashaqa	Lyfta: Gym Workout Tracker Log
**Domain One: Knowledge and Information**			
1.1 Customize and personalize some features	1	1	1
1.2 App created with expertise and/or information consistent with national guidelines	0	0	1
1.3 Ask for baseline information	1	1	1
1.4 Provide instruction on how to perform the behavior	1	1	1
1.5 Provide information about consequences of continuing and/or discontinuing behavior	0	1	0
**Total Domain**	**3**	**4**	**4**
**Domain Two: Goals and Planning**			
2.1 Ask for willingness for behavior change	0	0	1
2.2 Allow for the setting of goals	1	1	1
2.3 Review goals, update, and change when necessary	1	1	1
**Total Domain**	2	2	3
**Domain Three: Feedback and Monitoring**			
3.1 Understand the difference between current action and future goals	1	1	1
3.2 Allow the user to easily self-monitor behavior	1	1	1
3.3 Share behaviors with others (including social media or forums) and/or allow for social comparison	1	1	1
3.4 Give the user feedback—either from a person or automatically	1	1	1
3.5 Export data	1	0	1
3.6 Provide a material or social reward or incentive	1	1	1
3.7 Provide general encouragement	1	1	1
**Total Domain**	7	6	7
**Domain Four: Actions**			
4.1 Reminders and/or prompts or cues for activity	1	1	1
4.2 Encourage positive habit formation	1	1	1
4.3 Allow or encourage practice or rehearsal, and daily activities	1	1	1
4.4 Provide an opportunity to plan for barriers	1	0	0
4.5 Assist or suggest restructuring the physical or social environment?	0	0	0
4.6 Assists with distraction or avoidance	0	0	0
**Total Domain**	4	3	3
**Total App Score**	16	15	17

Responses were coded as follows: Present = 1; Absent = 0.

## Data Availability

The raw data supporting the conclusions of this article will be made available by the authors on request.

## Abbreviations

The following abbreviations are used in this manuscript:

MENA	Middle East and North Africa
ABACUS	App Behavior Change Scale
mHealth	mobile health
SFDA	Saudi Food and Drug Authority
UAE	United Arab Emirates

## Appendix A

Google form to evaluate eligible Apps in Saudi Arabia using ABACUS Framework.

Google form link: https://forms.gle/8Jwt6DhAQySjqBsN6 (accessed on 2 March 2026).

## Appendix B

**Table A1:** Arabic Wellness mHealth Apps Identified Across the MENA Region and Behavior Change Screening Results.

App Name	Country	Behavior Change
**Sehhaty** https://apps.apple.com/us/app/%D8%B5%D8%AD%D8%AA%D9%8A-sehhaty/id1459266578 (accessed on 28 January 2026)	Saudi Arabia	No
**StepsApp** https://apps.apple.com/sa/app/stepsapp-pedometer/id1037595083 (accessed on 28 January 2026)	Saudi Arabia, United Arab Emirates, Oman, Kuwait	Yes
**Rashaqa** https://play.google.com/store/apps/details?id=com.madarsoft.fitness&gl=EG (accessed on 28 January 2026)	Saudi Arabia, Egypt	Yes
**Wearfit Pro** https://apps.apple.com/sa/app/wearfit-pro/id1512947756 (accessed on 28 January 2026)	Saudi Arabia, Egypt, Algeria, Iraq, Yemen, Morocco, Jordan, United Arab Emirates, Libya, Lebanon, Oman	No
**Lyfta: Gym Workout Tracker Log** https://apps.apple.com/sa/app/lyfta-gym-tracker-planner/id6443740936 (accessed on 28 January 2026)	Saudi Arabia, Egypt, Algeria, Lebanon	Yes
**Sweatcoin** https://apps.apple.com/sa/app/sweatcoin-walking-step-tracker/id971023427 (accessed on 28 January 2026)	Saudi Arabia, Egypt, Algeria, Tunisia, Jordan, United Arab Emirates, Lebanon, Oman, Kuwait, Qatar, Bahrain	Yes
**Home workout—No equipments** https://apps.apple.com/eg/app/home-workout-no-equipments/id1313192037 (accessed on 28 January 2026)	Egypt, Algeria, Iraq, Yemen, Morocco, Tunisia, Jordan, United Arab Emirates, Libya, Lebanon, Oman, Qatar, Bahrain	Yes
**Lefun Health** https://apps.apple.com/eg/app/lefun-health/id1397285580 (accessed on 28 January 2026)	Egypt, Iraq	No
**Calz-Colorie Counter** https://apps.apple.com/eg/app/calz-calorie-counter-ai/id6738832996 (accessed on 28 January 2026)	Egypt, Iraq,	Yes
**ZTFit** https://apps.apple.com/eg/app/ztfit/id6472417706 (accessed on 21 January 2026)	Egypt, Iraq, Jordan, Lebanon, Oman	No
**Gym Workout Planner and Gym Log** https://apps.apple.com/eg/app/gym-workout-planner-gym-log/id1602190236 (accessed on 21 January 2026)	Egypt	Yes
**Welmi—Calorie Counter & Diet** https://apps.apple.com/us/app/welmi-calorie-counter-diet/id6741707862 (accessed on 21 January 2026)	Algeria, Morocco, Tunisia	Yes
**OKOK·International** https://apps.apple.com/dz/app/okok-international/id1028294311 (accessed on 21 January 2026)	Algeria, Iraq, Tunisia, Jordan, Lebanon	Yes
**FitCloudPro** https://apps.apple.com/iq/app/fitcloudpro/id1452851243 (accessed on 21 January 2026)	Iraq	Yes
**GloryFit** https://apps.apple.com/iq/app/gloryfit/id1237479843 (accessed on 21 January 2026)	Iraq	Yes
**Purecheck** https://apps.apple.com/ma/app/purecheck-scan-food-cosmetic/id1662348653 (accessed on 21 January 2026)	Morocco	No
**Open Food Facts** https://apps.apple.com/ma/app/open-food-facts-product-scan/id588797948 (accessed on 21 January 2026)	Morocco	No
**Eatwise** https://apps.apple.com/ae/app/calorie-counter-eatwise-ai/id6673907965 (accessed on 21 January 2026)	Tunisia, United Arab Emirates	Yes
**Mibro Fit** https://apps.apple.com/tn/app/mibro-fit/id1530759825 (accessed on 21 January 2026)	Tunisia	Yes
**Mi Fitness (Xiaomi Wear Lite)** https://apps.apple.com/tn/app/myfitnesspal-calorie-counter/id341232718 (accessed on 21 January 2026)	Tunisia, Jordan, United Arab Emirates, Oman, Kuwait, Qatar, Bahrain	Yes
**FittiCoin** https://apps.apple.com/jo/app/fitticoin/id1436858768 (accessed on 21 January 2026)	Jordan	Yes
**Pura by PureHealth** https://apps.apple.com/ae/app/pura-by-purehealth/id6449597603 (accessed on 21 January 2026)	United Arab Emirates	Yes
**Fere fit** https://apps.apple.com/ae/app/fere-fit/id1481346033 (accessed on 21 January 2026)	United Arab Emirates, Oman, Kuwait, Qatar	Yes
